# TRANSPARENT TESTA 16 and 15 act through different mechanisms to control proanthocyanidin accumulation in Arabidopsis testa

**DOI:** 10.1093/jxb/erx151

**Published:** 2017-05-10

**Authors:** W Xu, S Bobet, J Le Gourrierec, D Grain, D De Vos, A Berger, F Salsac, Z Kelemen, J Boucherez, A Rolland, G Mouille, J M Routaboul, L Lepiniec, C Dubos

**Affiliations:** 1 Institut Jean-Pierre Bourgin (IJPB), INRA, AgroParisTech, CNRS, Saclay Plant Sciences, Université Paris-Saclay, Versailles, France; 2 Genomic and Biotechnology of Fruit, UMR 990 INRA/INP-ENSAT, 24 Chemin de Borderouge-Auzeville, CS, Castanet-Tolosan Cedex, France; 3 Biochimie et Physiologie Moleculaire des Plantes (BPMP), INRA, CNRS, SupAgro-M, Université de Montpellier, Montpellier Cedex, France

**Keywords:** *Arabidopsis thaliana*, proanthocyanidins, seed coat, seed, tannins, testa, TT15, TT16

## Abstract

Flavonoids are secondary metabolites that fulfil a multitude of functions during the plant life cycle. In Arabidopsis proanthocyanidins (PAs) are flavonoids that specifically accumulate in the innermost integuments of the seed testa (i.e. endothelium), as well as in the chalaza and micropyle areas, and play a vital role in protecting the embryo against various biotic and abiotic stresses. PAs accumulation in the endothelium requires the activity of the MADS box transcription factor TRANSPARENT TESTA (TT) 16 (ARABIDOPSIS B-SISTER/AGAMOUS-LIKE 32) and the UDP-glycosyltransferase TT15 (UGT80B1). Interestingly *tt16* and *tt15* mutants display a very similar flavonoid profiles and patterns of PA accumulation. By using a combination of genetic, molecular, biochemical, and histochemical methods, we showed that both TT16 and TT15 act upstream the PA biosynthetic pathway, but through two distinct genetic routes. We also demonstrated that the activity of TT16 in regulating cell fate determination and PA accumulation in the endothelium is required in the chalaza prior to the globular stage of embryo development. Finally this study provides new insight showing that TT16 and TT15 functions extend beyond PA biosynthesis in the inner integuments of the Arabidopsis seed coat.

## Teaser

TT16 and TT15 independently control proanthocyanidin biosynthesis in the inner integuments of the Arabidopsis seed testa and their function extends beyond this tissue.

## Introduction

Flavonoids are secondary metabolites well known for the coloration of plant tissues that fulfil a multitude of functions during the plant life cycle, from protection against environmental stresses to modulation of plant growth and development (Lepiniec *et al.*, 2006; Grunewald *et al.*, 2012). In addition flavonoids are antioxidant molecules presenting beneficial properties for human health and are well known to impact the organoleptic, nutritive, and processing characteristics of feeds (Lepiniec *et al.*, 2006). In Arabidopsis seeds, flavonols and proanthocyanidins (PAs; also called condensed tannins) are the main flavonoids (Routaboul *et al.*, 2006, 2012). PAs specifically accumulate in the innermost integuments of the seed testa (i.e. endothelium) as well as in the chalaza and the micropyle areas. They play a crucial role in protecting the embryo against various biotic and abiotic stresses (Winkel-Shirley, 1998) and in modulating seed dormancy, longevity, and dispersion (Debeaujon *et al.*, 2000; Windsor *et al.*, 2000; Bueso *et al.*, 2014). Once oxidized, PAs confer a brown colour to mature seeds (Pourcel *et al.*, 2005) enabling the visual screening of mutants impaired in flavonoid accumulation named *transparent testa* (*tt*) (Shirley *et al.*, 1995).

PA biosynthesis is catalysed by a series of enzymes encoded by genes belonging to two main groups, the early biosynthetic genes (EBGs) and the late biosynthetic genes (LBGs) (Xu *et al.*, 2014*a*). The EBGs encode proteins whose activity provides precursors for the whole flavonoid biosynthetic pathway. The LBG group comprises genes encoding proteins involved in PA precursor biosynthesis as well as proteins involved in PA modification and compartmentalization (Appelhagen *et al.*, 2015). In addition to the EBGs and LBGs, *TT10*/*LAC15* encodes a LACCASE-type flavonoid oxidase involved in the oxidative polymerization of PAs (Pourcel *et al.*, 2005), and *TT15*/*UGT80B1* (UDP-GLUCOSE:STEROLGLUCOSYLTRANSFERASE) and *TT9*/*GFS9* encode proteins that are involved in vesicular trafficking controlling PA accumulation in the vacuole (Warnecke *et al.*, 1997; Focks *et al.*, 1999; DeBolt *et al.*, 2009; Ichino *et al.*, 2014). Interestingly, *TT15* has also been proposed to be the causative gene underlying a natural variation in PA accumulation occurring between the Col-0 and Cvi-0 Arabidopsis accessions (Routaboul *et al.*, 2012).

Genes involved in the transcriptional control of PA biosynthesis have also been characterized. For instance, specific R2R3-MYB (MYB123/TT2 and MYB5) and R/B-like bHLH (TT8/bHLH42, GL3/bHLH00 and EGL3/bHLH02; subgroup IIIf) transcription factors (TFs) together with TRANSPARENT TESTA GLABRA 1 (TTG1, WD repeat protein) form protein (MBW) complexes that specifically regulate LBG and *TT8* expression in a cell-specific manner leading to PA biosynthesis (Baudry *et al.*, 2004; Thévenin *et al.*, 2012; Xu *et al.*, 2013, 2014*a*, b; 2015). TTG2/DSL1/WRKY44 activity (which relies on TTG1 function) is required for integument cell elongation, mucilage biosynthesis and the production of PAs (Johnson *et al.*, 2002; Garcia *et al.*, 2005; Gonzalez *et al.*, 2009, 2016). It has also recently been proposed that TTG2 could act as an enhancer of MBW complex activities (Pesch *et al.*, 2014). *TT1*/*WIP DOMAIN PROTEIN 1* (*WIP1*) encodes a zinc finger TF that is thought to control seed coat development and to regulate the competency of endothelium cells to synthesize and accumulate PAs (Sagasser *et al.*, 2002; Appelhagen *et al.*, 2010, 2011).

*TT16*/*ABS*/*AGL32* (*ARABIDOPSIS B-SISTER*/ *AGAMOUS-LIKE 32*) encodes another TF involved in the transcriptional regulation of PA biosynthesis that belongs to the MADS box family (Shore and Sharrocks, 1995; Nesi *et al.*, 2002). MADS box TF can multimerize and form heterotrimeric complexes in order to regulate its target genes (de Folter *et al.*, 2006; Immink *et al.*, 2009). In plants, MADS box TFs were found to regulate development of organs such as flowers, ovules, seeds, leaves and roots and play an important role in the establishment of the Arabidopsis seed testa (Riechmann and Meyerowitz, 1997; Smyth, 2000; Ng and Yanofsky, 2001; Nesi *et al.*, 2002; Debeaujon *et al.*, 2003; de Folter *et al.*, 2006; Prasad and Ambrose, 2010; Prasad *et al.*, 2010; Mizzotti *et al.*, 2012, 2014; Ehlers *et al.*, 2016; Ezquer *et al.*, 2016; Figueiredo *et al.*, 2016; Xu *et al.*, 2016). The expression of *TT16* orthologous genes is restricted in angiosperm and gymnosperm species to female reproductive organs, mainly the integuments of the ovules (Becker *et al.*, 2002; Nesi *et al.*, 2002; Kaufmann *et al.*, 2005; Tonaco *et al.*, 2006; Deng *et al.*, 2012; Chen *et al.*, 2013; Rhodes *et al.*, 2014). Unlike most of the other regulatory *tt* mutants, *tt16* accumulates PAs in the chalaza and micropyle areas while they are absent from the endothelium (Nesi *et al.*, 2002). It is noteworthy that both the flavonoid profile and the pattern of PA accumulation displayed by the *tt16* mutant are similar to those of *tt15* suggesting that both genes may act on the same genetic pathway (Nesi *et al.*, 2002; Routaboul *et al.*, 2006, 2012; DeBolt *et al.*, 2009). Interestingly, *TT2* ectopic expression restores PA accumulation in *tt16* indicating that TT16 acts upstream of the PA biosynthetic pathway, and that the ability of the cells to accumulate PA is not directly dependent on TT16 activity (Nesi *et al.*, 2002). The lack of TT16 activity is also associated with ectopic cell divisions leading to disorganized and irregularly shaped PA-accumulating cells (Nesi *et al.*, 2002). This phenotype is exacerbated in the *tt16 stk*/*agl11* (*seedstick*) double mutant (Mizzotti *et al.*, 2012). These results demonstrate that TT16 is involved in the transcriptional control of endothelium development. More recently, TT16 function (together with GORDITA, GOA/AGL63, its closest homologue) was associated with nucellus degeneration following ovule fertilization (Erdmann *et al.*, 2010; Xu *et al.*, 2016). In addition, TT16 together with SHATTERPROOF 1 and 2 (SHP1/AGL1 and SHP2/AGL5, two closely related MADS-box TFs) was shown to be involved in the control of endosperm development and in the coordination of cell divisions in ovule integuments and seed coat development (Ehlers *et al.*, 2016). Several studies have shown that TT16 can interact *in vivo* with various members of the MADS box protein family, suggesting that TT16 may be involved in the transcriptional control of additional facets of seed coat development (de Folter *et al.*, 2005; Kaufmann *et al.*, 2005; Tonaco *et al.*, 2006; Immink *et al.*, 2009). In canola, another member of the Brassicaceae, beside the exhibition of abnormal endothelium development and decreased PA content, the expression of most genes known to be involved in the PA biosynthetic pathway, as well as several related genes such as *TTG2*, was significantly reduced in *Bntt16* mutant lines compared with wild-type plants (Deng *et al.*, 2012; Chen *et al.*, 2013).

In order to refine our understanding of the roles and mode of action of TT16 and TT15 during seed development and PA biosynthesis, a combination of genetic, molecular, biochemical, and histochemical methods was used. We demonstrated that TT16 and TT15 act upstream of the PA biosynthetic pathway through two distinct genetic pathways. We then demonstrated that the activity of TT16 in regulating cell fate and PA accumulation in the endothelium is required prior to the globular stage in the chalaza area. Finally this study showed that TT16 and TT15 activities extend beyond PA biosynthesis in the endothelium, as TT16 most probably regulates the fate of the inner integuments of the testa, whereas TT15 plays a role at the whole plant level.

## Materials and methods

All PCRs were carried out using high-fidelity Phusion DNA polymerase, according to the manufacturer’s instructions (Thermo Scientific Finnzymes). PCR products were subsequently sequenced after recombination or cloning into their destination vectors. All the primers used in this study are described in Supplementary Table S1 at *JXB* online. Expression analyses (qRT-PCR) were performed as described in Dubos *et al.* (2008).

### Plant material

Arabidopsis accession Wassilewskija (WS) was used as wild-type control. The mutant lines *tt16-1* (*dxt32*; Nesi *et al.*, 2002) and *tt15-2* (*cob16*; Routaboul *et al.*, 2012) were obtained from the Versailles Biological Resource Centre (http://publiclines.versailles.inra.fr). The double *tt15 tt16* mutant was obtained by crossing the *tt15-2* and *tt16-1* alleles. Plants expressing β-glucuronidase (GUS) under the control of the *TT8* and *TT15* promoters are described in Xu *et al.* (2013, 2014*a*). All methods and conditions used for plant growth, plant transformation, and selection for transgenic lines were as previously reported by Nesi *et al.* (2000).

### Studied Arabidopsis gene IDs

The Arabidopsis gene IDs were as follows: *BANYULS*/*ANR*, At1g61720; *CHS/TT4*, At5g13930; *TT2*/*MYB123*, At5g35550; *TT8*/*bHLH042*, At4g09820; *TT15*/*UGT80B1*, At1g43620; *TT16*/*AGL32/ABS*, At5g23260.

### Proanthocyanidin staining and measurement

PA staining of 4-day-old seeds was carried out using vanilline reagent as described in Debeaujon *et al.* (2003). Quantitative PA measurements were carried out on 15 mg of dried seeds accordingly to Routaboul *et al.* (2012) using methanol–acetone–water–trifluoroacetic acid (30/42/20/0.05, v/v/v/v) to maximize PA extraction. Samples were measured in triplicates in two independent biological repetitions.

### Constructs

Fusions of *TT16* (*pTT16*: 1597 bp prior to the ATG) and *BAN* (*pBAN*: 236 bp prior to the ATG) promoters to the Gateway^TM^ recombination cassette were carried out as described for the promoter of *TT8* (1.5 kb prior to the ATG) in Dubos *et al.* (2008). Briefly, *pTT16* and *pBAN* were PCR-amplified from genomic DNA (WS) with the pTT16-5′-HindIII/pTT16-3′-XbaI and pBAN-5′-HindIII/pBAN-3′-XbaI primer pairs, respectively. The obtained DNA fragments were subsequently cloned into the pBIB-Hyg-GTW vector (Dubos *et al.*, 2008) digested with *Hin*dIII and *Xba*I, giving the pBIB-Hyg *pTT16*:GTW and *pBAN*:GTW vectors.

*TT2* and *TT15* coding sequences (CDSs) were PCR-amplified from WS cDNAs using the cTT2-B1/cTT2-B2 and cTT15-B1/cTT15-B2 primer pairs, respectively. Genomic *TT16* (*gTT16*), which corresponds to the DNA sequence between the ATG and stop codons, including both exons and introns, was PCR-amplified from genomic DNA (WS) using the cTT16-gATG-B1/cTT16-gSTOP-B2 primer pair. The same primers were also used to amplify from WS cDNA cTT16L/ABSI (long: 759 bp) and cTT16S/ABSII (short: 744 bp) CDSs corresponding to the two splice variants of *TT16*. The obtained DNA fragments were then BP-recombined into the pDONR207 vector (Gateway^TM^).

*TT2* and *TT15* CDSs were recombined into the pMDC32 vector (Curtis and Grossniklaus, 2003) for overexpression (which contains two copies of the *35S* minimal promoter from the cauliflower mosaic virus, *p70S*).

*cTT16L*/*ABSI* and *cTT16S*/*ABSII* CDSs were LR-recombined into the *pTT16*:GTW vector.

*gTT16* was LR-recombined into *pTT16*:GTW, *pTT8*:GTW, *pBAN*:GTW, and pMDC32 vectors.

TT2 promoter (2 kb prior to the ATG) for GUS analysis was cloned into pDONR207 and then LR-recombined into pGWB3 (Nakagawa *et al.*, 2007).

For each construct, 6–12 independent transgenic plants were analysed, and representative observations are presented.

### Histochemical detection of GUS activity

GUS staining for seeds expressing *promoter:uidA* gene fusion constructs were performed as described in Berger *et al.* (2011). For each construct, 6–12 independent transgenic plants were analysed, and representative observations are presented.

### Immunofluorescence labelling of cell wall

Four-day-old siliques were collected on ice (extremities were removed, and the remaining part was cut into two pieces) and incubated for 1 h at 4 °C in the fixation buffer (1× PBS (Eurobio), 2% formaldehyde, and 0.1% triton X-100) after vacuum treatment (three times). Samples were then dehydrated using a series of increasing ethanol concentration in PBS (30%, 50%, 70%, 90%, 100%) at 4 °C (2 h each). Siliques were then stained using toluidine blue (0.01% in absolute ethanol), and transferred into a 2:1 followed by a 1:1 absolute ethanol–wax (wax: PEG400–1-hexadecanol, 9:1) solution for 2 h at 40 °C each, and finally transferred into a 1:2 solution (overnight at 40 °C). Samples were then incubated twice for 3 h in 100% wax solution at 40 °C before polymerization. Cross sections of 8 μm were finally cut using a Leica RM2165 microtome, and sample ribbons were placed on a drop of sterile water (Versol) on polyethylene slides, and left to dry overnight at 37 °C.

Immunolabelling using the JIM4 and JIM8 monoclonal antibodies (PlantProbes, Leeds, UK) was carried out as described in Macquet *et al.* (2007). Samples were then observed using a confocal microscope (Leica TCS-SP2 AOBS, Leica Microsystems). Spectral bands from 498 to 567 nm were selected in order to specifically detect the Alexa Fluor 488 fluorescence.

## Results

### TT15 acts upstream of the PA biosynthetic pathway

**Fig. 1.**
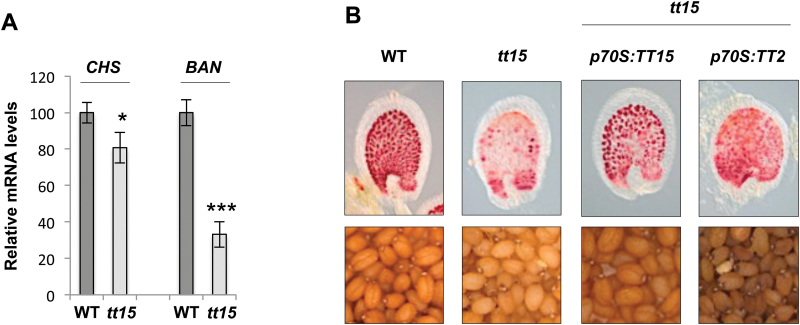
*TT15* acts upstream the PA biosynthetic pathway. (A) *tt15 transparent testa* phenotype is associated with a decrease in expression of PA biosynthetic genes, such as *CHS* (an EBG) and *BAN* (an LBG). mRNA steady state levels of *CHS* and *BAN* genes in wild-type (WT) and *tt15* 4-day post-fertilization siliques was measured by quantitative RT-PCR. Measurements are expressed as the percentage of the WT level. *t*-Test significant difference: *P<0.05 and ***P<0.001. Error bars show SE. (B) *tt15* complementation assays demonstrate that overexpression of *TT15* and *TT2* CDSs is sufficient to restore *tt15 transparent testa* phenotypes. Whole-mount vanillin (vanilaldehyde) staining of seeds (globular stage, 4 d post-fertilization) from WT and *tt15* lines overexpressing *TT15* and *TT2* CDSs. Following vanillin treatment in acidic conditions, PAs give a bright red product.

The two mutants *tt16* and *tt15* share a very similar pattern of flavonoid accumulation. They both have lower content of the major accumulated flavonols (quercitrin and quercetin-3-rhamnoside) and very little PAs (Routaboul *et al.*, 2006, 2012) when compared with wild-type (WT) seeds. Both *tt16* and *tt15* mutants accumulate PA mostly in the chalaza and micropyle areas. Expression analysis (quantitative RT-PCR) showed that the *tt15* had decreased PA accumulation strongly correlated with a decrease of *BAN* mRNA levels (and to a lesser extent of *CHS*, the first EBG of the PA biosynthetic pathway) (Fig. 1A and Table 1), which was similar to what was previously observed in *tt16* developing seeds (Nesi *et al.*, 2002; Debeaujon *et al.*, 2003). To confirm that TT15 acts upstream the PA biosynthetic pathway, like *TT16*, the *TT2* coding sequence (CDS) was overexpressed in *tt15* (using the *p70S* promoter). As a positive control for this complementation assay, overexpression of the *TT15* CDS was also carried out using the same promoter. In both cases PA accumulation increased in immature and mature transgenic seeds, indicating that, similarly to TT16, TT15 functions upstream of the PA biosynthetic pathway (Fig. 1B and Table 1).

**Table 1.** *Analysis of mature seed PA content (soluble and insoluble PAs) in wild-type, tt16, tt15, and transgenic lines used for* tt16 *and* tt15 *complementation assays* Proanthocyanidin content is expressed as mg cyanidin g^–1^ seed.

Line	Soluble	Insoluble
WT	5.97 ± 0.02	3.52 ± 0.04
*tt16*	0	0.28 ± 0.01
*p70S:gTT16* in *tt16*	12.05 ± 0.06	4.07 ± 0.01
*p70S:TT2* in *tt16*	0.92 ± 0.10	1.22 ± 0.05
*pTT16:TT16L* in *tt16*	0	0.17 ± 0.01
*pTT16:TT16S* in *tt16*	0	0.16 ± 0.02
*p70S:TT15* in *tt16*	0	0.17 ± 0.02
*tt15*	0	0.34 ± 0.01
*p70S:TT15* in *tt15*	4.34 ± 0.11	2.00 ± 0.01
*p70S:TT2* in *tt15*	1.56 ± 0.02	1.64 ± 0.01
*p70S:TT16* in *tt15*	0	0.43 ± 0.03

### TT15 and TT16 affect PA accumulation in an independent manner

**Fig. 2.**
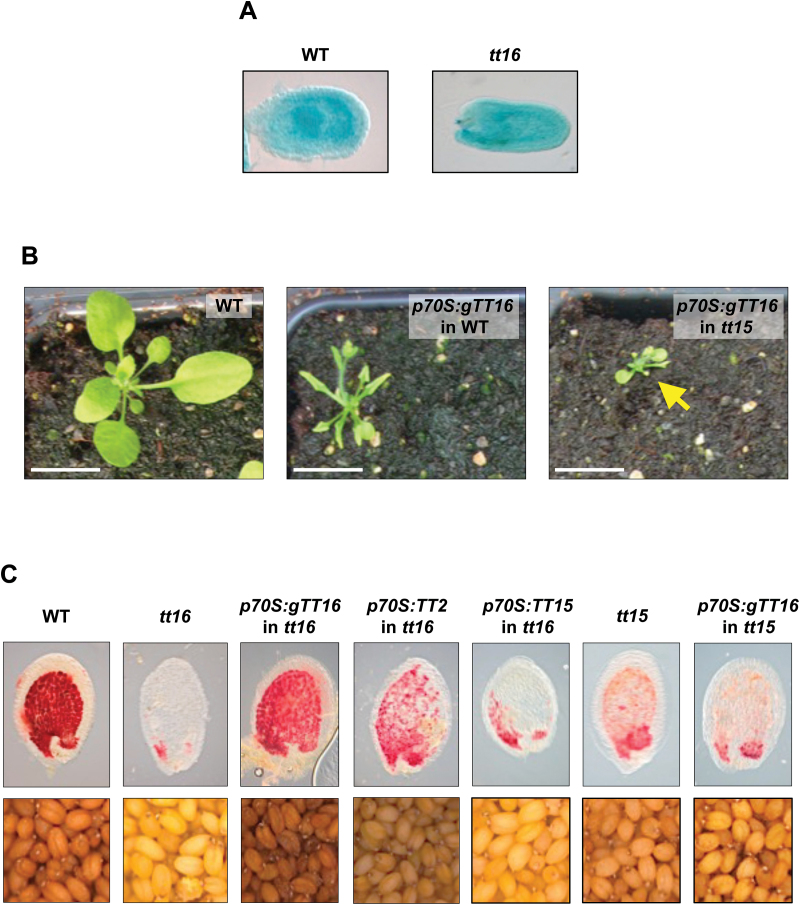
*TT15* and *TT16* affect PA accumulation through two independent genetic pathways. (A) Pattern of *TT15* promoter activity in wild-type (WT) and *tt16* seeds (globular stage) revealed by the detection of GUS activity. (B) Effect of *gTT16* overexpression on WT and *tt15* vegetative tissues highlighting that growth defects (stunted plants with curly leaves and reduced flower size; Nesi *et al.*, 2002) are enhanced in *tt15* mutants when compared with WT plants. (C) Cross-complementation experiments in which *gTT16* (the genomic sequence comprising the introns and exons present between the start and stop codons of *TT16* as *TT16* is expressed under two spliced variants, *TT16L*/*ABSI* and *TT16S*/*ABSII*; Nesi *et al.*, 2002) and *TT15* CDSs were overexpressed in *tt15* and *tt16*, respectively. *gTT16* and *TT2* CDSs were overexpressed in *tt16* in order to confirm the functionality of the DNA fragments that were used and the downstream position of TT2 when compared with TT16 in the PA biosynthetic pathway, respectively (Nesi *et al.*, 2002; Debeaujon *et al.*, 2003). Upper panels: 4-day-old (globular stage) seeds treated with vanilline reagent, which specifically stains PAs red (whole mount); lower panels: seed colour.

**Fig. 3.**
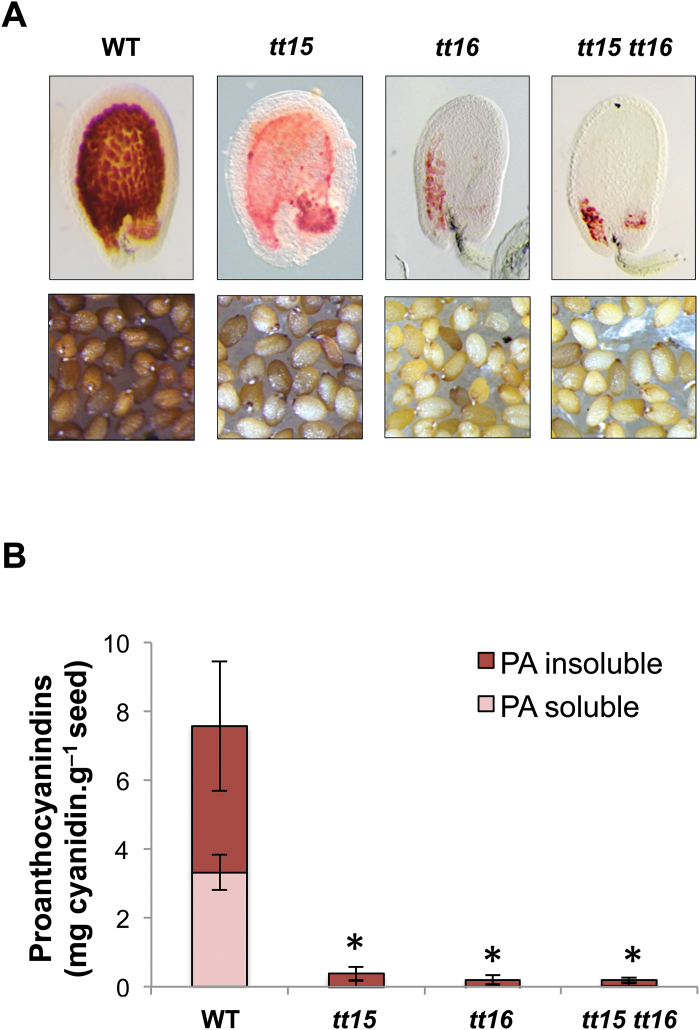
Arabidopsis *tt15 tt16* double mutant seed characterization. The *tt15 tt16* double mutant displays a *transparent testa* phenotype that is similar to that of *tt16* (i.e. no additive effect) confirming that TT15 and TT16 act through two different routes to modulate PA accumulation in seeds. (A) Upper panels: 4-day-old (globular stage) seeds treated with vanilline reagent, which specifically stains PAs red (whole mount); lower panels: seed colour. (B) Mature seed PA content analysis (soluble and insoluble PAs). *t*-Test significant difference: *P<0.001. Error bars show SE.

**Fig. 4.**
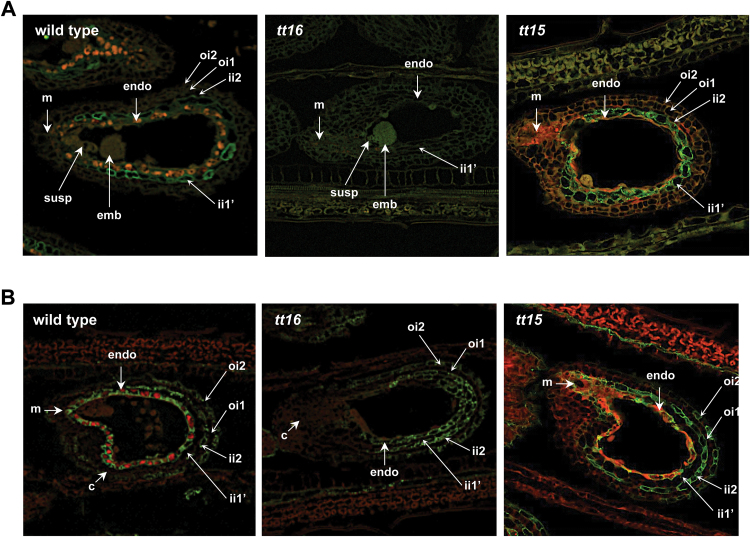
Arabidopsis seed immunohistolabelling using monoclonal antibodies targeting specific arabinogalactan-proteins present in the cell wall of the testa. Cross section of wild-type, *tt16* and *tt15* seeds (globular stage) were labelled using (A) JIM4 antibody (targeting the ii1′ cell layer) and (B) JIM8 antibody (targeting the ii1/endothelium, ii2, and oi2 cell layers). Bright green: fluorescence associated with the JIM4 and JIM8 antibodies; bright red: PA fluorescence. c, chalaza; ii, inner integument; emb, embryo; endo, endothelium; m, micropyle; oi, outer integument; susp, suspensor.

Despites the above-described similarities, contrary to *tt16*, the endothelium cell shape of *tt15* does not seem to be affected, suggesting that TT15 does not function upstream of TT16 (Nesi *et al.*, 2002; Debeaujon *et al.*, 2003). Conversely, we found that the activity of *TT15* promoter was unaffected in *tt16*, when compared with WT seeds (Fig. 2A). Rather, the phenotypic defects observed in vegetative tissues upon *gTT16* overexpression (i.e. stunted plants with curly leaves, reduced flower size and shrunken siliques; Nesi *et al.*, 2002) were accentuated in *tt15*, suggesting that the two proteins act in different genetic pathways (Fig. 2B and Supplementary Fig. S1). To investigate this hypothesis further, cross-complementations of *tt16* and *tt15* were carried out. For this purpose the *TT16* genomic region (*gTT16*) and *TT15* CDS were overexpressed (*p70S* promoter) in *tt15* and *tt16*, respectively (Fig. 2C). Although both constructs were able to complement their respective mutants (see previous paragraph for *TT15* CDS; Fig. 1B), no cross-complementation was observed, indicating that the function of each gene does not rely on the function of the other (Fig. 2C and Table 1). It is noteworthy that both *gTT16* and *TT2* CDS were able to complement *tt16* when overexpressed with the same promoter (*p70S*; Figs 2C and 4, Supplementary Fig. S2, and Table1), confirming the functionality of the DNA fragments used and the position of TT2 downstream of TT16 in the PA biosynthetic pathway (Nesi *et al.*, 2002; Debeaujon *et al.*, 2003). These data also confirm that the competency of PA-accumulating cells to synthetized and accumulate PA remain conserved in *tt16* mutant seeds. Lastly, we found that the *tt15 tt16* double mutant displayed a *transparent testa* phenotype that was similar to that of *tt16* (i.e. no additive effect), confirming that TT15 and TT16 act through distinct genetic routes (Fig. 3).

### TT16 activity extends beyond the control of the sole endothelium development

In order to further characterize the different cellular impacts of *tt16* and *tt15* mutations, immunohistolabelling experiments were carried out. The rationale was that in Arabidopsis seeds the ii1 (PA-accumulating cells—endothelium) and the ii1′ (parenchymatous cells) cell layers are derived from the ovular endothelium through periclinal divisions (Debeaujon *et al.*, 2003); the formation of these two cell layers is achieved once the ovule becomes mature. Two markers were used for these experiments, namely the JIM4 and JIM8 monoclonal antibodies that target specific arabinogalactan-proteins present in the cell wall of different cell layers of the testa. Markers of the cell wall have been selected because various studies have demonstrated that cell wall properties can vary between different cell type and in response to developmental signals (Cassab, 1998; Freshour *et al.*, 2003). The JIM4 antibody specifically marked the ii1′ cell layer in WT seed coat (Fig. 4A). Interestingly, no JIM4 labelling was observed in *tt16* seeds, in contrast to what was observed in *tt15* seeds (Fig. 4A). It is noteworthy that JIM4 labelling was restored in *tt16* seeds overexpressing *gTT16*, but not in *tt16* seeds overexpressing *TT2* or *TT15* CDS (Supplementary Fig. S2A). JIM8 antibody specifically labelled three cell layers of the testa in WT seeds, namely the ii1 (endothelium, PA-accumulating cells), inner integument 2 (ii2) and outer integument 2 (oi2, mucilage-producing cells) cell layers (Fig. 4B). This labelling pattern was also observed for the *tt15* mutant as well as for *tt16* lines overexpressing *gTT16* (Fig. 4B and Supplementary Fig. S2B). JIM8 labelling of the ii1, ii2 and oi2 cell layers was conserved in *tt16* seed coat, but extended to the ii1′ cells including the cells issued from ectopic divisions (Debeaujon *et al.*, 2003) (Fig. 4B and Supplementary Fig. S2B). Similar cell labelling was also observed when *TT2* or *TT15* CDSs were overexpressed in *tt16* (Supplementary Fig. S2B).

Together these observations support that TT16 participates in cell fate determination of both daughter cell layers of the ovular endothelium, namely ii1 and ii1′.

### PA accumulation in the endothelium requires TT16 activity prior to the globular stage

**Fig. 5.**
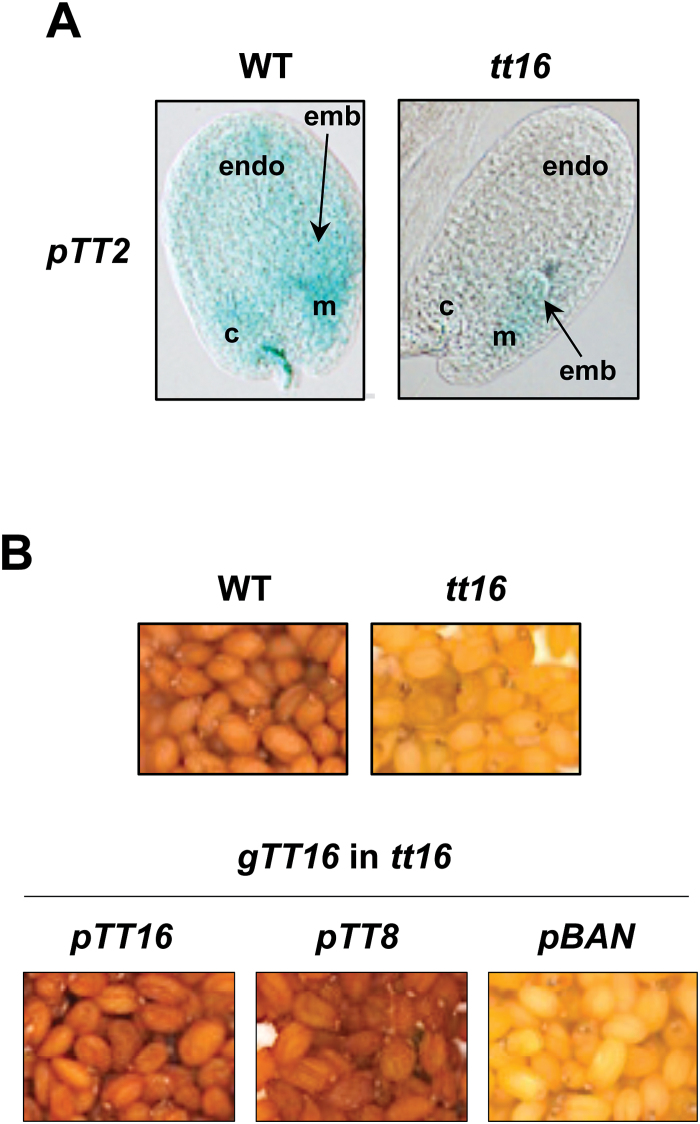
PA accumulation in the endothelium of Arabidopsis seeds requires TT16 activity in the chalaza area early during ovule development. (A) Pattern of *TT2* promoter activity in wild-type and *tt16* seeds at the globular stage, the developmental stage that coincides with the maximum of *BAN* (LBG) expression in the seed testa (Debeaujon *et al.*, 2003). (B) Complementation of *tt16 transparent testa* phenotype using the genomic sequence comprising the introns and exons present between the start and stop codons of *TT16* (*gTT16*) as *TT16* is expressed under two spliced variants (*TT16L*/*ABSI* and *TT16S*/*ABSII* CDSs, which are 759 and 744 bp long, respectively; Nesi *et al.*, 2002). When expressed under its own promoter, *gTT16* is sufficient to complement *tt16* phenotypes. Complementation of *tt16* was also observed when the promoter of *TT8* was used whereas no complementation occurred with the *BAN* promoter. c, chalaza; emb, embryo; endo, endothelium; m, micropyle.

The main MBW complex involved in PA biosynthesis is composed of TT2 (R2R3-MYB), TT8 (bHLH) and TTG1 (Baudry *et al.*, 2004; Thévenin *et al.*, 2012; Xu *et al.*, 2014*a*, 2013). *TT2* and *BAN* (the first LBG whose activity is specific to PA biosynthesis) expression starts prior to fertilization in the micropyle area and then propagates throughout the PA-accumulating cells upon fertilization (Debeaujon *et al.*, 2003). Interestingly we found that *TT2* expression remains restricted to the micropyle region in the *tt16* mutant indicating that TT16 activity is required for the progression of *TT2* expression within the PA-accumulating cells (Fig. 5A). *TT8* expression in *tt16* seeds is also lower in the endothelium (ii1) when compared with WT seeds (Xu *et al.*, 2013).

To investigate if there was a developmental time frame in which TT16 activity was required for proper PA biosynthesis and accumulation in seeds, two different promoters were used to drive the expression of *TT16* for *tt16* complementation experiments (Fig. 5B). For this purpose the promoters of *TT8* (*pTT8*) and *BAN* (*pBAN*) were used, as the expression of the corresponding genes in PA-accumulating cells initiates at distinct developmental stages (Nesi *et al.*, 2000; Debeaujon *et al.*, 2003; Supplementary Fig. S3). Because *TT16* is expressed under two alternative mRNA forms, the genomic sequence comprising the introns and exons present between the start and stop codons was used (*gTT16*). We first successfully complemented the *transparent testa* (*tt*) phenotype of *tt16* using the *TT16* promoter (*pTT16*, ~1.6 kb prior to the start codon), confirming that the genomic DNA fragment containing the *TT16* CDS was functional (Fig. 5B). We observed a similar *tt16* complementation when *pTT8* was used, but not with *pBAN* (Fig. 5B). This observation suggests that there is a developmental frame in which PA-accumulating cells can perceive signals associated with TT16 activity (i.e. prior to the globular stage). In addition data gathered on *pTT16:gTT16:GUS* (~1.6 kb) and *pTT8:GUS* activities (restricted to the chalazal area in WT and *tt16*, respectively; Xu *et al.*, 2013, 2016) and *TT8* expression (initiating from the chalazal area prior to fertilization; Supplementary Fig. S3) demonstrated that the expression of *TT16* in the chalaza area prior to the globular stage was sufficient to trigger PA accumulation. In support of this conclusion it was also recently shown that when *pTT16* (~1.6 kb) is fused to the *uidA* reporter gene, no GUS activity is observed in seeds, whereas the expression of *TT16* in PA-accumulating cells requires the regulatory sequences that are present up to 3.4 kb prior to the *TT16* start codon (Ehlers *et al.*, 2016; Xu *et al.*, 2016).

### *TT16* genomic sequence spanning the CDS region plays a key role in the control of PA accumulation in seeds

**Fig. 6.**
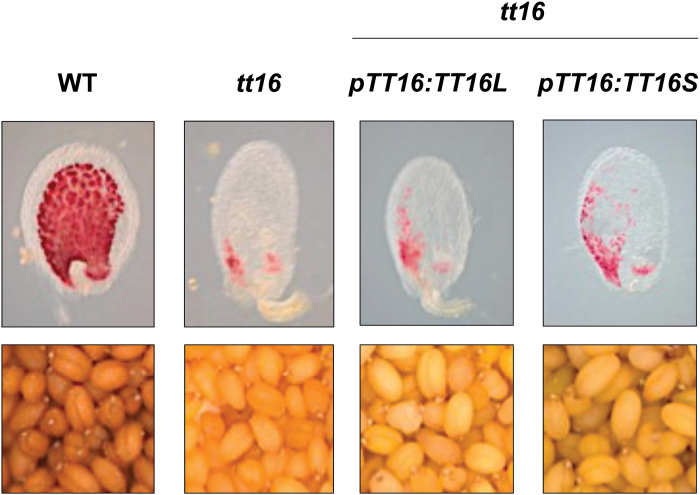
*tt16* complementation assays using both splice variants of *TT16*. Neither of the two *TT16* splice variant CDSs (*TT16L*/*ABSI* and *TT16S*/*ABSII*, which are 759 and 744 bp long, respectively) was sufficient to revert the absence of PAs in *tt16* endothelium. Upper panels: 4-day-old (globular stage) seeds treated with vanilline reagent, which specifically stains PAs red (whole mount); lower panels: seed colour.

In Arabidopsis *TT16* is alternatively spliced into two CDSs that are 759 bp (*TT16L*/*ABSI*) and 744 bp (*TT16S*/*ABSII*) long (Nesi *et al.*, 2002). In another member of the Brassicaceae, *Brassica napus* (canola), four homologues of the Arabidopsis TT16L variant (i.e. BnTT16.1 to BnTT16.4) displaying between 75 and 80% identity at the protein level were identified and characterized; each of them complements the *tt16* mutation when ectopically expressed using the CaMV *35S* promoter (Chen *et al.*, 2013). To determine if the genomic region of *TT16* plays a role in regulating *TT16* expression, we carried out *tt16* complementation assays in which *TT16L* and *TT16S* CDSs as well as *gTT16* were expressed under the control of *pTT16*. Immature 4-day-old (globular stage) seeds of WT, *tt16*, and *tt16* transgenic lines were thus treated with vanilline reagent, which specifically stains PAs red (Fig. 6), and then the PA content was measured in dry seeds (Table 1). PA-accumulating cells (i.e. endothelium, chalaza, and micropyle) of WT seeds were stained red, whereas only the chalaza and micropyle areas were stained in *tt16* seeds. This observation correlated with the strong decrease in PA content measured in *tt16* dry seeds when compared with WT (Routaboul *et al.*, 2006). Surprisingly, although the expression of *gTT16* complements the *tt16* mutant, no complementation was observed when *TT16L* or *TT16S* CDS was expressed under the control of *pTT16*. (Figs 5B and 6). These data are fully consistent with previous results showing the importance of *TT16* genomic sequence for the correct expression of the gene (Xu *et al.*, 2016).

## Discussion

**Fig. 7.**
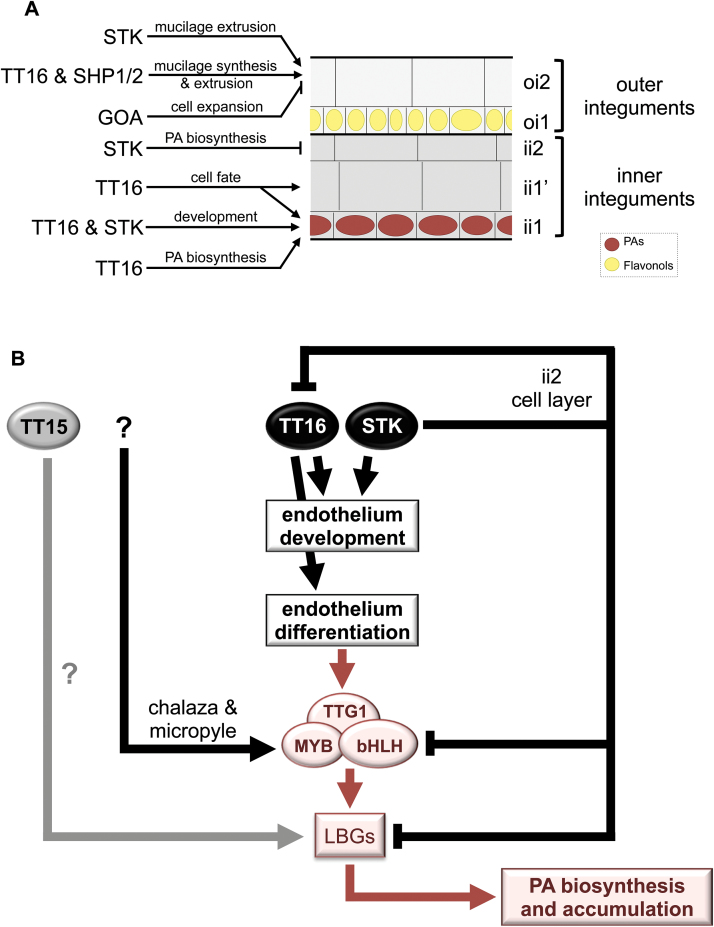
Schematic representation of the roles played by TT16 and TT15 in the control of PA biosynthesis and accumulation and/or testa cell fate determination. (A) The testa of the Arabidopsis seed is composed of five cell layers, three inner integuments (ii1, ii1′, and ii2) and two outer integuments (oi1 and oi2). ARABIDOPSIS B-SISTER/AGAMOUS-LIKE 32 (TT16/ABS/AGL32) is well known for its role in the control of PA biosynthesis and accumulation in the endothelium (ii1 cell layer). Endothelium development requires both TT16 and SEEDSTICK/AGL11 (STK). STK is a repressor of PA biosynthesis in the ii2 cell layer. The closest homologue of TT16, GORDITA/AGL63 (GOA; Erdmann *et al.*, 2010), is a repressor of cell elongation in the oi2 cell layer. The oi2 cell layer is also the site for mucilage synthesis and extrusion that is affected by the STK, SHATTERPROOF 1 (SHP1), SHP2 and TT16 activities (Ehlers *et al.*, 2016; Ezquer *et al.*, 2016). We confirmed in this study that TT16 is a key regulator of cell fate of the two cell layers that derive from the ovular endothelium through periclinal divisions, namely ii1 (PA-accumulating cells, endothelium) and ii1′ (parenchymatous cells). (B) PAs specifically accumulate in the endothelium (ii1 cell layer). STK inhibits PA accumulation in the ii2 cell layer by modifying the chromatin state of key regulatory genes (e.g. the bHLH transcription factors *TT8* and *EGL3* involved in the formation of MBW complexes, or *TT16*) and biosynthetic genes (e.g. the LBG *BAN*) involved in this pathway (Mizzotti *et al.*, 2014). STK and TT16 redundantly regulate endothelium development (Mizzotti *et al.*, 2012). Nevertheless, TT16 solely is involved in the differentiation of the endothelium into a PA-accumulating cell layer (Nesi *et al.*, 2002; Mizzotti *et al.*, 2012). Interestingly, even if STK is expressed early during ovule and seed development in PA-accumulating cells, STK expression does not inhibit the accumulation of PAs. The molecular mechanisms that counterbalance STK inhibitory effect on PA biosynthesis in these tissues are still to be identified. Similarly, the molecular mechanism by which TT15 regulates PA biosynthesis and accumulation in the endothelium also remains to be characterized. Finally, how PA biosynthesis and accumulation occur independently of TT16 and TT15 in the chalaza and micropyle areas is still unknown as well. LBGs: late biosynthetic genes; MBW complexes: MYB–bHLH–TTG1 (WD40 repeat protein). (This figure is available in colour at *JXB* online.)

Previous analyses have demonstrated that *tt16* and *tt15* mutants share a very similar pattern of flavonoid accumulation in the seed coat (Routaboul *et al.*, 2006, 2012). Both mutants have altered accumulation of PA in the endothelium, whereas PAs still accumulate in the chalaza and micropyle areas (Fig. 2). In this study, we showed that TT15, like TT16, acts upstream the PA biosynthetic pathway, but through a distinct genetic route (Fig. 7). Interestingly, we also found that the growth defects triggered by *gTT16* overexpression in vegetative tissues were accentuated in *tt15*, indicating that TT15’s function extends beyond PA accumulation in seeds to whole plant development (Fig. 2B and Supplementary Fig. S1; DeBolt *et al.*, 2009).

TT16 is a key regulator of endothelial cell fate; however it was still unclear if its role within the testa extended beyond the development of this cell layer. In order to explore this we used for immunolabelling experiments two different markers targeting the cell wall of distinct cell layers of the seed testa (Fig. 4 and Supplementary Fig. S2). These markers were the JIM4 (the ii1′ cell layer) and JIM8 (PA-accumulating cells and the ii2 and oi2 cell layers) monoclonal antibodies directed against different arabinogalactan epitopes. Through this approach we confirmed that TT16 is involved in the transcriptional control of cell fate determination of the two most inner integuments (ii1 and ii1′) of the seed testa and that the cells issued from ectopic divisions derived from the ii1 cell layer (Nesi *et al.*, 2002). In support of this finding it was recently shown that TT16 and SHATTERPROOF 1 and 2 (SHP1/AGL1 and SHP2/AGL5) play an antagonistic role in the control of ii1′ cell layer development (Ehlers *et al.*, 2016). The mucilage extrusion defects observed in *tt16* or *tt16 shp1 shp2* mutants when compared with WT seeds indicate that TT16 function in testa development is broader than primarily thought (Ehlers *et al.*, 2016). The fact that the alteration of TT16 activity impacts cell wall properties of the ii1′ cell layer suggests that TT16 may, like STK, play a role in the control of structural and mechanical properties of the seed testa (Ezquer *et al.*, 2016). In contrast, no difference was observed between WT and *tt15* seeds confirming the idea that TT16 and TT15 functions are independent. Altogether these data demonstrate that *TT15* and *TT16* are involved in PA accumulation in the endothelium through different pathways, and that their functions extend beyond this tissue.

With the aim to get new insights into the role that TT16 plays during seed development we investigated if there was a developmental time frame in which TT16 activity was required for proper PA biosynthesis and accumulation. Complementation experiments of *tt16* were carried out by expressing *gTT16* under the control of three different promoters that are active at distinct developmental stages in PA-accumulating cells (i.e. *pTT16*, *pTT8*, and *pBAN* genes). This strategy revealed that TT16 activity is required in the chalaza area prior to the globular stage of embryo development for proper endothelium development and PA accumulation (Fig. 1). Moreover, based on *TT16* expression data (Mizzotti *et al.*, 2012; Xu *et al.*, 2016) and the pattern of *pTT16*, *pTT8*, and *pBAN* activity in Arabidopsis seeds (Supplementary Fig. S1; Debeaujon *et al.*, 2003; Xu *et al.*, 2013) it is likely that TT16 activity in the chalaza area prior to fertilization is sufficient to trigger endothelium cell fate determination.

While studying the potential role of *TT16* spliced variants (*TT16L*/*ABSI*, 759 bp, and *TT16S*/*ABSII*, 744 bp) during seed development, we confirmed that a *TT16* genomic sequence spanning the CDS region plays a key role in the control of PA accumulation in seeds (Fig. 2). In addition, and unlike what has been found in canola (*Brassica napus*), we found that *TT16L* (as well as *TT16S*) was not sufficient to restore *tt16* mutant phenotypes (Chen *et al.*, 2013). Taken together these observations suggested that the intronic regions of *TT16* are necessary for the initiation of PA biosynthesis in the endothelium and must thus contain key regulatory sequences. The importance of the intragenic sequences for the control of gene expression has already been reported for other MADS box TFs. This is for example the case for *AGAMOUS* (*AG*), a key transcriptional regulator of floral organ specification, for which most of the regulatory elements that control its expression are located in the second intron of the gene (Kaufmann *et al.*, 2010). Whether or not regulatory sequences are located within the introns of *TT16* will need further investigation. Another important question that remains to be addressed is determining the role that TT16S plays during seed development, in particular in the light of the work of Chen *et al.* (2013) mentioned above. Is the dimerization between TT16L and TT16S previously reported (de Folter *et al.*, 2005) necessary for TT16 activity? This is, for example, the case with STK/AGL11 (SEEDSTICK; Kaufmann *et al.*, 2005), another MADS box TF that is partially redundant with TT16 in regulating endothelium development (Mizzotti *et al.*, 2012; Fig. 7). Close petunia homologues of TT16 (FLORAL BINDING PROTEIN 24; FBP24) and STK (FBP11) were also shown to interact *in vivo* in yeast and in plant cells (Tonaco *et al.*, 2006). Taken together these data could suggest that part of TT16’s activity relies on the formation of (dimeric or trimeric) protein complexes involving TT16 and STK.

Nevertheless, with respect to PA accumulation in seeds, TT16 and STK present antagonistic activities. TT16 promotes PA accumulation in the endothelium (Nesi *et al.*, 2002) whereas STK inhibits the expression of genes involved in PA biosynthesis and accumulation, as well as those such as *TT8*, *EGL3* and *TT16* encoding regulatory proteins in the inner integument 2 (ii2) (Mizzotti *et al.*, 2014). Interestingly, the presence of STK in the chalaza and micropyle areas in mature ovules does not inhibit the accumulation of PA, whether TT16 is present or not (Debeaujon *et al.*, 2003; Xu *et al.*, 2013; Mizzotti *et al.*, 2014). This observation suggests that an additional regulatory mechanism controls the expression of PA biosynthetic genes in the chalaza and micropyle areas, being dominant over STK inhibition and independent of TT16 induction (Fig. 7). TT16 could also repress the deposition of STK-dependent repressive marks on the chromatin of genes involved in PA biosynthesis (Mizzotti *et al.*, 2014), or conversely, TT16 may facilitate the deposition of chromatin marks associated with an active chromatin state at these loci.

*TT16* and *GOA/AGL63* (*GORDITA*) are paralogous genes (Erdmann *et al.*, 2010). To date, GOA function has been associated with the control of fruit size, through the modulation of cell expansion (Prasad and Ambrose, 2010; Prasad *et al.*, 2010). GOA and TT16 have additive roles in seed coat development as revealed by the phenotype of the *goa tt16* double mutant whose seeds display phenotypic defects associated with each mutation; long and narrow oi1 cells for *goa* seeds and flat and irregularly shaped ii1 cells that lack PA accumulation for *tt16* seeds (Prasad *et al.*, 2010). One of the hypotheses associated with this observation would be that both genes play a similar role in cell fate determination, but in different cell layers because of distinct promoter activities (Prasad and Ambrose, 2010), and through different molecular mechanisms. This later assumption is supported by the fact that GOA possesses a specific protein–protein interaction domain (the ‘deviant’ domain, DD) that results in the absence of shared protein interaction partners with the other ABS proteins (Erdmann *et al.*, 2010). Recently, it was demonstrated that TT16 acts redundantly with GOA to promote nucellus degeneration upon fertilization leading to the formation and correct positioning of the chalazal endosperm (Xu *et al.*, 2016). Nevertheless, the role that GOA plays in the chalaza area remains to be elucidated (Prasad *et al.*, 2010).

PA biosynthesis and accumulation in Arabidopsis seeds is regulated at the transcriptional level by various MYB–bHLH–WDR (MBW) protein complexes (Baudry *et al.*, 2004; Thévenin *et al.*, 2012; Xu *et al.*, 2013, 2014*a*, *b*, 2015). Similar MBW complexes have been shown to be involved in cell fate determination of trichomes and root hairs. Cell-to-cell movement of proteins involved in these MBW complexes was found to be central in this process (Bernhardt *et al.*, 2005; Balkunde *et al.*, 2011). PA accumulation in seeds initiates in the chalaza and micropyle areas before spreading throughout the whole endothelium, following the expression pattern of *TT2* and *TT8* (Debeaujon *et al.*, 2003; Xu *et al.*, 2013). In contrast, PA accumulation in *tt16* is restricted to the chalaza and micropyle areas, as is the case for *TT2* expression (Fig. 1). Similarly, the expression of *TT8* in *tt16* is unaffected in the chalaza and micropyle areas whereas its spreading throughout the endothelium is delayed (Xu *et al.*, 2013; Supplementary Fig. S1). Because the overexpression of *TT2* is sufficient to overcome the *transparent testa* phenotype of *tt16*, it could be hypothesized that cell-to-cell communication from the chalaza and micropyle areas toward the endothelium is central for initiating PA biosynthesis in the endothelium. Nevertheless, it cannot be excluded that such a signal could derive from other seed tissues (e.g. ii1′ cell layer, endosperm) in response to TT16 activity. In addition a recent study has highlighted the importance of auxin production in the endosperm to initiate the development of the Arabidopsis seed coat, a process involving another MADS box TF, AGL62 (Figueiredo *et al.*, 2016). Indeed, these hypotheses would require additional experiments in order to be validated, and necessitate identifying the signal (e.g. MBW protein members). The identification and characterization of genes that are directly regulated by *TT16* in the seed testa will be the next challenge in order to clearly understand how *TT16* activity synchronizes cell fate determination, PA accumulation and seed development.

## Supplementary data

Supplementary data are available at *JXB* online.

Fig. S1. Growth defects in vegetative tissues (stunted plants with curly leaves and reduced flower size) due to *gTT16* overexpression are enhanced in *tt15* mutants when compared with wild-type plants.

Fig. S2. Arabidopsis seed immunohistolabelling using monoclonal antibodies targeting specific arabinogalactan-proteins present in the cell wall of the testa, in *tt16* and *tt15* complementation experiments.

Fig. S3. Pattern of *TT8* promoter activity in developing wild-type ovules and seeds revealed by the detection of GUS activity.

Table S1. Primers used in this study.

## Supplementary Material

Supplementary_Table_S1

Supplementary_Figures_S1_S3
