# Supplementary material for: TRANSPARENT TESTA 16 and 15 act through different mechanisms to control proanthocyanidin accumulation in Arabidopsis testa
Source: J Exp Bot. 2017 May 10;68(11):2859–70. doi: 10.1093/jxb/erx151 (PMC5853933; doi:10.1093/jxb/erx151)
Supplement: Supplementary_Table_S1 [file erx151_suppl_supplementary_table_s1.pdf]

Table S1: Primer used in this study

Name	Sequence	Purpose
**pTT16-5'-HindIII**	ACGAAGCTTttcagtgttcgagtttcagc	Cloning of *TT16* (1597 bp) promoter
**pTT16-3'-XbaI**	GTTCTAGActctctcttcttcctcttatgagtgg	Cloning of *TT16* (1597 bp) promoter
**pBAN-5'-HindIII**	ACGAAGCTTTTGGTAGATGATAACAAATC	Cloning of *BAN* (236 bp) promoter
**pBAN-3'-XbaI**	GTTCTAGAGATTGTACTTTTGAAATTAC	Cloning of *BAN* (236 bp) promoter
**pTT2-B1**	ggggacaagtttgtacaaaaaagcaggctTCcgatattctatcgcaacatgg	Cloning of *TT2* (2065 bp) promoter
**pTT2-B2**	ggggaccactttgtacaagaaagctgggtctctcacttttctctctcttgtgg	Cloning of *TT2* (2065 bp) promoter
**cTT2-B1**	ggggacaagtttgtacaaaaaagcaggctTCATGGGAAAGAGAGCAACTAC	Cloning of *TT2* cds
**cTT2-B2**	ggggaccactttgtacaagaaagctgggtcTCAACAAGTGAAGTCTCGGAGCC	Cloning of *TT2* cds
**cTT15-B1**	ggggacaagtttgtacaaaaaagcaggctTCATGGCTAGTAATGTATTTGATC	Cloning of *TT15* cds
**cTT15-B2**	ggggaccactttgtacaagaaagctgggtcTCACACGCCACCACATGGAAG	Cloning of *TT15* cds
**cTT16-gATG-B1**	ggggacaagtttgtacaaaaaagcaggcttcATGGGTAGAGGGAAGATAG	Cloning of *gTT16*, and *TT16L* and *TT16S* cds
**cTT16-gSTOP-B2**	ggggaccactttgtacaagaaagctgggtcTTAATCATTCTGGGCCGTTGG	Cloning of *gTT16*, and *TT16L* and *TT16S* cds

NB: Primers used for *BAN* and *CHS* qRT-PCR are described in Xu *et al*., 2014a
