# Supplementary material for: TRANSPARENT TESTA 16 and 15 act through different mechanisms to control proanthocyanidin accumulation in Arabidopsis testa
Source: J Exp Bot. 2017 May 10;68(11):2859–70. doi: 10.1093/jxb/erx151 (PMC5853933; doi:10.1093/jxb/erx151)
Supplement: Supplementary_Figures_S1_S3 [file erx151_suppl_supplementary_figures_s1_s3.pdf]

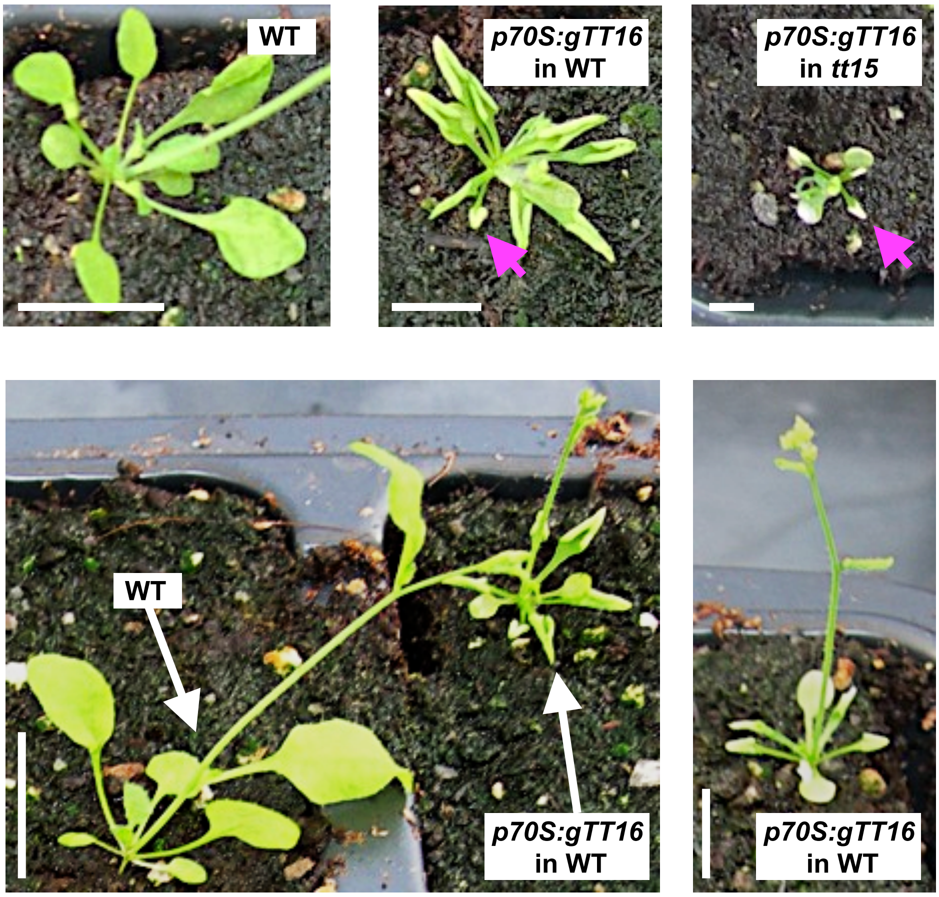

Fig. S1. Growth defects in vegetative tissues (stunted plants with curly leaves and reduced flower size) due to *gTT16* overexpression are enhanced in *tt15* mutants when compared to wild type plants.

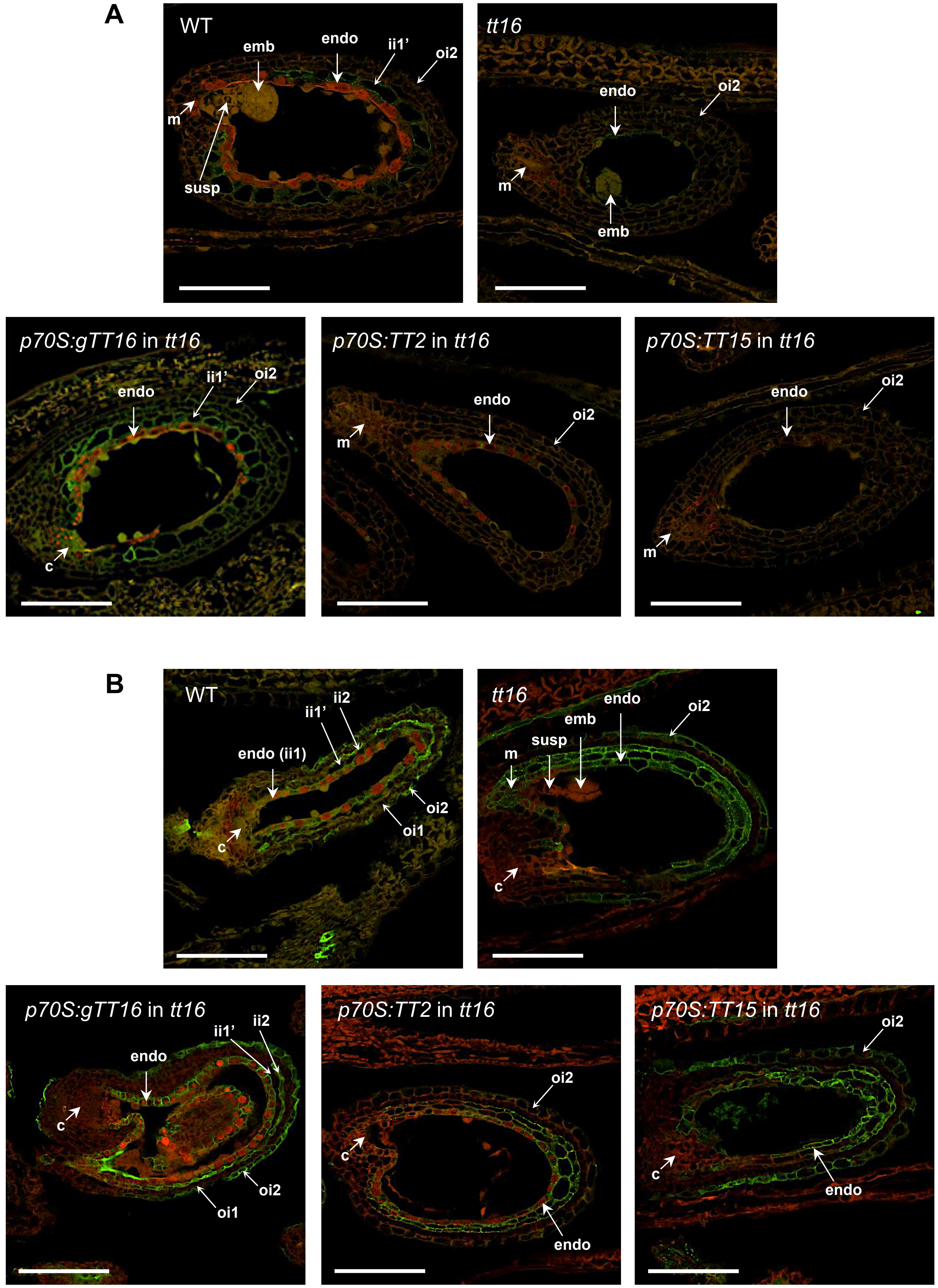
A
WT
emb
endo
ii1’
oi2
m
susp
tt16
endo
oi2
m
emb
p70S:gTT16 in tt16
ii1’
oi2
endo
c
p70S:TT2 in tt16
endo
oi2
m
p70S:TT15 in tt16
endo
oi2
m
B
WT
ii2
ii1’
endo (ii1)
oi2
oi1
c
tt16
endo
oi2
emb
susp
m
c
p70S:gTT16 in tt16
ii2
ii1’
endo
c
oi2
oi1
p70S:TT2 in tt16
oi2
c
endo
p70S:TT15 in tt16
oi2
c
endo

Fig. S2. ***Arabidopsis thaliana*** **seeds i*mmunohistolabeling* using monoclonal antibodies targeting specific arabinogalactan-proteins present in the cell wall of the *testa*, in *tt16* and *tt15* complementation experiments.** Cross section of wild type, *tt16* and *tt15* seeds (globular stage) and complementaed lines were labelled using the **(A)** JIM4 (targeting the ii1' cell layer) and **(B)** JIM8 (targeting the ii1/endothelium, ii2 and oi2 cell layers) antibodies. Bright green: fluorescence associated with the JIM4 and JIM8 antibodies, bright red: PAs fluorescence. c: chalaza, m: micropyle, endo: endothelium, susp: suspensor, emb: embryo, ii: inner integument, oi: outer integument.

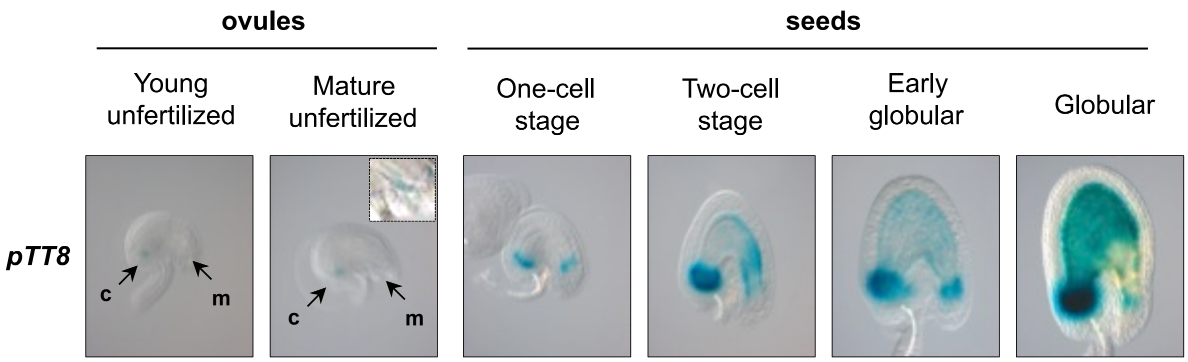

Fig. S3. Pattern of *TT8* promoter activity in developing wild type ovules and seeds revealed by the detection of GUS activity.
